# Temporal trends in surgical treatment of inflammatory bowel disease following introduction of biological drugs in Norway and Sweden

**DOI:** 10.1136/bmjgast-2025-001828

**Published:** 2025-06-19

**Authors:** Amanda Högdén, Vera Perrin, Hans-Olov Adami, Mette Kalager, Tine Jess, Weimin Ye, Jessica Young, Lise Mørkved Helsingen, Erle Refsum, Johannes Blom

**Affiliations:** 1 Department of Clinical Science and Education, Karolinska Institute, Stockholm, Sweden; 2 Department of Surgery, Södersjukhuset, Karolinska Institute, Stockholm, Sweden; 3 Department of Medical Epidemiology and Biostatistics, Karolinska Institutet, Stockholm, Sweden; 4 Clinical Effectiveness Research Group, University of Oslo, Oslo, Norway; 5 Clinical Effectiveness Research Group, Institute of Health and Society, University of Oslo, Oslo, Norway; 6 Clinical Effectiveness Research Group, Oslo University Hospital, Oslo, Norway; 7 Center for Molecular Prediction of Inflammatory Bowel Disease, Department of Clinical Medicine, Aalborg Universitet, Copenhagen, Denmark; 8 Department of Gastroenterology and Hepatology, Aalborg Universitetshospital, Aalborg, Denmark; 9 Medical Epidemiology and Biostatistics, Karolinska Institute, Stockholm, Sweden; 10 Institute of Environmental Medicine, Unit of Epidemiology, Karolinska Institute, Stockholm, Sweden; 11 University of Oslo Faculty of Medicine, Oslo, Norway; 12 Oslo University Hospital, Oslo, Norway

**Keywords:** INFLAMMATORY BOWEL DISEASE, ULCERATIVE COLITIS, SURGERY FOR IBD, CROHN'S DISEASE

## Abstract

**Objective:**

The advent of biological drugs has revolutionised management of inflammatory bowel disease (IBD). However, the extent to which these novel pharmacological drugs have reduced the need for surgical treatment remains incompletely quantified.

We aimed to investigate the risk of first, major surgery in IBD in a population-based, large epidemiological study.

**Methods:**

We empanelled a cohort comprising all 85 974 patients diagnosed with ulcerative colitis (UC) and 42 760 with Crohn’s disease (CD) in Norway and Sweden in 1987 through 2017. We used log-rank tests to compare the cumulative probability of surgical treatment for UC and CD. Using multivariable Cox proportional hazards models, we estimated hazard ratios (HR) with 95% CIs by year of diagnosis, age, sex and extent of disease.

**Results:**

During a mean follow-up of 9.9 years, surgery was undertaken in 11 187 (13.0%) patients with UC (12.3 per 1000 person-years) and in 11 307 (26.4%) patients with CD (30.0 per 1000 person-years). In UC, the cumulative 5-year probability of surgery decreased from 16.2% in patients diagnosed in 1987–1994 to 5.8% in those diagnosed in 2011–2017 (p<0.001). In CD, the corresponding decline was from 30.1% to 13.9% (p<0.001). In multivariable analyses, the likelihood of surgical treatment decreased during the study period by 61% (HR 0.39, 95% CI 0.36 to 0.42) in UC and by 31% (HR 0.69, 95% CI 0.65 to 0.75) in CD.

**Conclusions:**

Following the introduction of biologic drugs, the need for surgical treatments has been dramatically reduced in patients with UC and moderately reduced in patients with CD.

**WHAT IS ALREADY KNOWN ON THIS TOPIC**

- Previous studies suggest that surgical rates in inflammatory bowel disease (IBD) declined after the introduction of biological treatment. However, many of these studies are small and not strictly population-based.

**WHAT THIS STUDY ADDS**

- With a population-based design, large size, and virtually complete follow-up in nationwide high-quality registers, this study provides reliable evidence regarding trends in the surgical management of IBD over 30 years.
- This study finally provides high-quality data of the decreasing trend of surgical treatment in IBD. While the trend is evident and persistent for ulcerative colitis, it seems to have haltered in CD after the millennium.

**HOW THIS STUDY MIGHT AFFECT RESEARCH, PRACTICE OR POLICY**

- Trends of treatments provide important information for further research as well as for policy-makers.

## INTRODUCTION

 Both ulcerative colitis (UC) and Crohn’s disease (CD)—the two main forms of inflammatory bowel disease (IBD)—are associated with onset at young ages and often lifelong chronic inflammation of the bowel. These features, along with increasing incidence, particularly in newly industrialised countries, and decreasing mortality due to more effective medical treatment, cause an increase in the prevalence of IBD globally.^1^ Hence, optimised management is an important priority not only for individual patients but also for the public health system.^2 3^

In recent decades, introduction of biologic drugs has revolutionised management of IBD,^4^ motivating new guidelines that promote intense and proactive ‘top-down’ pharmacologic treatment.^5^,^9^ Notwithstanding this progress, many patients both with UC^10^ and CD^11^ need surgical treatment because they do not respond sufficiently, although a biological drug that has previously failed can be reconsidered after surgery.^12^ Surgery also remains a cornerstone in the acute treatment of IBD.^13 14^

Several studies^315^,^29^ but not all^30^,^35^ have shown a decrease in rates of surgery in recent decades. Because these studies were predominantly small, covered short time periods, were not population-based and/or did not separately analyse UC and CD, the extent to which novel drugs have reduced the need for surgical treatment remains uncertain. Our primary aim was to provide robust evidence of the trends in surgical treatment in patients with UC and CD. Additionally, we wanted to investigate the types of surgical procedures and the indication for surgery. To this end, we undertook a longitudinal population-based study allowing detailed analyses of temporal trends during three decades in rate and type of surgery separately in patients with UC and CD.

## METHODS

### Setting

The objective of the study was to investigate the trends of surgical treatment in IBD. This was done through the Scandinavian research collaboration calledIBD-Scandinavian CANcer in IBD study I-SCAN, (I-SCAN), evaluating outcomes of all patients in Norway and Sweden diagnosed with UC or CD since 1987.^36^,^38^ These two neighbouring Scandinavian countries are highly homogenous with regards to ethnicity, sociodemographic characteristics, and access to population registers as well as structure of the public healthcare system with virtually no private inpatient care. The individually unique national registration number assigned to all residents in Norway and Sweden enables linkage to these high-quality and virtually complete population registers containing data on morbidity and surgical procedures (see below), mortality (the Norwegian Cause of Death Register and the Swedish Cause of Death Register), and migration (Norway: the national population register; Sweden: the total population register).

### Study population

In Norway, we empanelled the analytic cohort based on relevant International Classification of Disease (ICD) codes for IBD collected from all public hospitals from 1987 to 2015, containing information on both inpatient and outpatient care. In Sweden, we used data from the inpatient register (with a nationwide coverage since 1987) from 1987 to 2017 and also information from the outpatient register included from 2001 to 2017.^39^ Patients aged <10 or >100 at the first registered IBD diagnosis were excluded.

To ensure high specificity of IBD diagnosis, we required at least two inpatient and/or outpatient diagnosis codes of IBD, which yields a positive predictive value of an IBD diagnosis of 90% in our cohort.^37^ We used the ICD codes from the first two visits to define IBD subtypes at baseline: patients were classified as having UC (ICD-9: 556; ICD-10: K51) or CD (ICD-9: 555; ICD-10: K50) if their first two codes were UC or CD, respectively, and unclassified IBD (IBD-U) if their first codes were indeterminate colitis (ICD10: K523) or any combination of codes (online supplemental table 1). The date of the first IBD diagnosis defined the start of follow-up.

### Surgery

The outcomes of interest were major abdominal surgeries, defined by procedure codes. In both countries, the inpatient register and hospital data provided information about the surgical procedures by registered procedure codes. These codes were extracted from the Norwegian and Swedish version of the NOMESCO Classification of Surgical Procedures.^40^ We focused on major abdominal surgeries, including proctectomies, colectomies and intestinal resections. Surgical procedures were divided by type and anatomical site of resection (online supplemental tables 2 and 3). Only surgery after the date of first IBD diagnosis was included.

We used the ICD codes registered at the date of surgery as a proxy for the indication for surgery. We grouped relevant ICD codes into IBD, benign tumours, cancer in situ, obstruction (including ileus), mesenteric thromboembolism, functional intestinal disorders, fissures/fistulas, peritonitis or acute inflammation, bleeding, and stomal dysfunction (online supplemental table 4).

### Statistical analysis

We first calculated cumulative probability of major surgery using Kaplan-Meier survival analysis and group differences were assessed using the log-rank test. Follow-up started at the first diagnosis of IBD (UC, CD) and ended at the date of first surgery, date of diagnosis of colorectal cancer (CRC), emigration, death or at the end of follow-up, whichever occurred first.

To assess changes in surgical treatment by year of first diagnosis, the cohort was divided into patients diagnosed in 1987–1994, 1995–2002, 2003–2010, and 2011–2017. We calculated the cumulative probability of surgery at 1, 5 and 10 years after diagnosis in these groups (for patients diagnosed in 2011–2017, cumulative probability at 7 years was calculated). Patients were also analysed separately for UC, CD or IBD-U. Incidence rates of surgery per 1000 patient-years at <1, 1–5, 6–10, and >10 years were calculated for each type of IBD. We used univariate and multivariate Cox proportional hazards model to calculate HRs with 95% CI of major surgery during follow-up, for UC and CD separately. The main exposure was year of diagnosis (groups 1987–1994, 1995–2002, 2003–2010 and 2011–2017). The multivariable regression model was adjusted for age, sex, nationality, cumulative extent of disease (UC), cumulative behaviour of disease (CD) and perianal disease (CD) (online supplemental table 5). We used log-minus-log curves and Schoenfeld residuals to investigate the proportional hazards assumption. Statistical significance of multiplicative interaction was assessed by the likelihood ratio test (p<0.001) comparing models with and without cross-product interaction terms between year of diagnosis and country as the potential effect modifier.

The trends of indication for surgery (ICD codes registered at the date of surgery were used as a proxy for indication) were calculated as a proportion by dividing the number of registered diagnoses, by the total number of major abdominal surgeries for each year.

Analyses were performed using SAS software V.9.4 (Copyright 2016 SAS Institute Cary, North Carolina) and Stata V.18 software (StataCorp. 2023. *Stata Statistical Software: Release 18*. College Station, Texas: StataCorp LLC). The reporting follows the Strengthening the Reporting of Observational studies in Epidemiology checklist (online supplemental material).

## RESULTS

### Descriptive characteristics

The analytic cohort included 141 109 patients with at least two diagnostic listings of IBD (online supplemental figures 1 and 2). A total of 85 974 (60.9%) patients had UC, 42 760 (30.3%) CD and 12 375 (8.8%) IBD-U. The proportion of men was slightly higher than women in UC (53.2% vs 46.8%) and lower in CD (47.6% vs 52.4%). Mean age at first diagnosis was 43.9 years (18.5 SD) in UC, 40.1 years (18.8 SD) in CD and 42.9 years (20.0 SD) in IBD-U (table 1).

**Table 1:** Patient baseline characteristics

Number of subjects	Norwaynumber (%)	Swedennumber (%)	Totalnumber (%)
Total IBD cohort	46 495	94 614	141 109
Male	23 690 (51.0)	48 519 (51.3)	72 209 (51.2)
Female	22 805 (49.0)	46 095 (48.7)	68 900 (48.8)
UC	28 664	57 310	85 974
Male	15 233 (53.1)	30 512 (52.4)	45 745 (53.2)
Female	13 431 (46.9)	26 798 (47.6)	40 229 (46.8)
CD	13 634	29 126	42 760
Male	6473 (47.5)	13 877 (47.6)	20 350 (47.6)
Female	7161 (52.5)	15 249 (52.4)	22 410 (52.4)
IBD-U	4197	8178	12 375
Male	1984 (47.3)	4130 (50.5)	6114 (49.5)
Female	2213 (52.7)	4048 (49.5)	6261 (50.6)
Age at diagnosis	Mean (±SD)	Mean (±SD)	Mean (±SD)
	Number (%)	Number (%)	Number (%)
Full cohort	42.2 years (18.4)	42.9 years (19.0)	42.6 years (18.8)
<20	4868 (10.5)	10 738 (11.4)	15 606 (11.1)
≥20–<40	18 598 (40.0)	35 368 (37.4)	53 966 (38.3)
≥40–<60	13 977 (30.1)	28 156 (29.8)	42 133 (29.9)
≥60	9052 (19.5)	20 352 (21.5)	29 404 (20.8)
UC	44.0 (18.0)	43.9 (18.7)	43.9 (18.5)
CD	38.1 (18.1)	41.0 (19.1)	40.1 (18.8)
IBD-U	43.2 (19.4)	42.8 (20.3)	42.9 (20.0)
Numbers and cases by year of diagnosis			Number
UC			
1987–1994 (surgeries, %)			15 913 (4234, 26.6%)
1995–2002 (surgeries, %)			25 246 (3712, 14.7%)
2003–2010 (surgeries, %)			25 574 (2397, 9.4%)
2011–2017 (surgeries, %)			19 241 (844, 4.3%)
Full cohort (surgeries, %)			85 974 (11 187, 13.0%)
CD			
1987–1994 (surgeries, %)			8847 (4315, 48.7%)
1995–2002 (surgeries, %)			10 687 (3374, 31.6%)
2003–2010 (surgeries, %)			12 533 (2509, 12.0%)
2011–2017 (surgeries, %)			10 693 (1109, 10.4%)
Full cohort (surgeries, %)			42 760 (11 307, 26.4%)

CD, Crohn’s disease; IBD, inflammatory bowel disease; IBD-U, unclassified inflammatory bowel disease; UC, ulcerative colitis.

Time between first and second registered ICD code of IBD was less than 1 year for 72.1% of the patients (online supplemental figure 3). The total follow-up time included 1 395 459 person-years with a mean follow-up of 9.9 years, median of 8.5 years and a maximum of 31 years.

The cumulative extent of disease among patients with UC was 15.7% with proctitis, 16.7% with left-sided colitis, 48.1% with extensive disease, and 19.3% were undefined. In CD, the location of the disease was terminal ileum in 22.3%, colon in 20.6%, 42.0% had ileocolonic involvement, and 14.2% were not defined. In CD, 74.3% had no stricturing or penetrating disease, 16.5% had stricturing disease, 5.9% had penetrating disease with fistulation, and 3.3% had both penetrating and stricturing disease.

**Figure 1:**
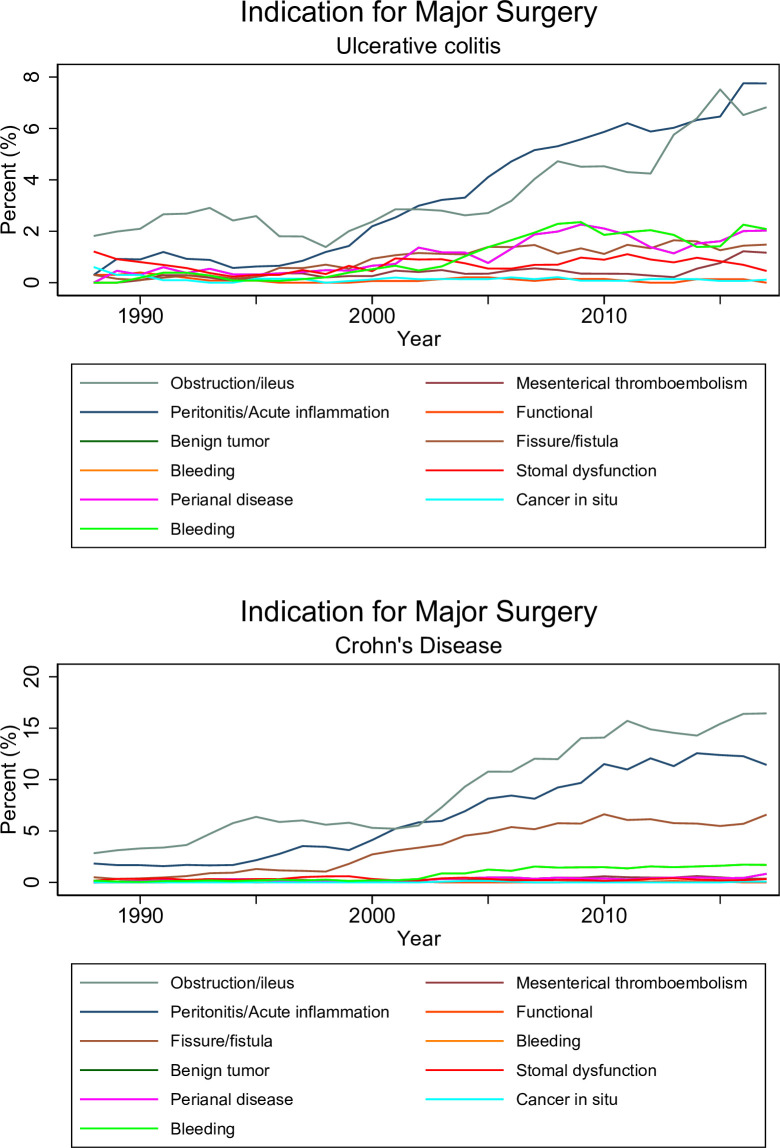
Indication for major surgery in patients with ulcerative colitis and Crohn’s disease. ICD codes registered at the date of surgery were used as a proxy for indication. Percentage of procedures with specific diagnoses, by year. IBD diagnosis excluded. Smoothed curve over average of 3 years. For IBD-U, see online supplemental figure 4. IBD, inflammatory bowel disease; IBD-U, unclassified IBD; ICD, International Classification of Disease.

Major surgery was undertaken in 11 187 patients with UC (12.3 per 1000 person-years), 11 307 with CD (30.0 per 1 000 person-years), and 1 761 with unclassified IBD-U (table 1 and online supplemental tables 6–8). A total of 4679 patients had their first surgery at the same date as the date of IBD diagnosis. The dominating indication for surgery (ICD codes registered at the date of surgery were used as a proxy for indication) was the underlying disease accounting for 79% in UC and 87% in CD. In both UC and CD, the second most common indication for surgery was obstruction/ileus (figure 1, IBD-U, see online supplemental figure 4).

### Time trends

In both patient groups, the rate of surgery per 1000 patients decreased over the study period. In patients with UC, 38.5 surgeries per 1000 patients were performed in 1990, compared with 5.6 per 1000 patients in 2017. The most common procedure in UC was total colectomy, followed by proctocolectomy (online supplemental table 9 and figure 5). In CD, there were 82.4 surgeries per 1000 in 1990, compared with 11.2 per 1000 in 2017. In CD, the most common procedure was ileocaecal resection, followed by small bowel resection (online supplemental table 9 and figure 6).

With some exceptions, the most common surgical procedure remained stable over the years. The most frequent procedure was total colectomy in UC and ileocaecal resection in CD. However, in patients with UC, there was a shift in 2015, when distal colonic resection replaced proctocolectomy as the second most common procedure. In CD, proximal colonic resection was the second most procedure between 1997 and 2015 when small bowel resection was once again the second most common procedure (online supplemental table 9, figures 5 and 6). The mean age at first surgical procedure was 47.7 years (SD 18.3) among patients with UC, and 42.9 years (SD 17.5) in patients with CD. In both patient groups, the mean age at surgery increased throughout the study period (online supplemental table 10).

In patients with UC, the overall cumulative probability of major surgery was 4.4% at 1 year, 9.5% at 5 years, and 12.9% at 10 years after first diagnosis. The cumulative 5-year probability of surgery decreased over time from 16.2% in patients diagnosed in 1987–1994 to 5.8% in those diagnosed in 2011–2017 (table 2). In patients with CD, the overall cumulative probability of major surgery was 10.4%, 20.0% and 27.1% at 1, 5 and 10 years, respectively (table 2). The 5-year cumulative probability of surgery decreased over time from 30.1% in patients diagnosed in 1987–1994 to 13.9% in those diagnosed in 2011–2017 (table 2).

**Table 2:** Cumulative probability of surgery at 1, 5 and 10 years in ulcerative colitis, Crohn’s disease and unclassified IBD

Cumulative probability	1 year % (95% CI)	5 years % (95% CI)	10 years % (95% CI)
Ulcerative colitis	4.4 (4.3 to 4.5)	9.5 (9.3 to 9.7)	12.9 (12.7 to 13.2)
1987–1994	8.0 (7.6 to 8.4)	16.2 (15.7 to 16.8)	21.5 (20.9 to 22.2)
1995–2002	4.8 (4.6 to 5.1)	9.8 (9.4 to 10.1)	12.8 (12.3 to 13.2)
2003–2010	3.1 (2.9 to 3.3)	7.3 (6.9 to 7.6)	9.7 (9.4 to 10.1)
2011–2017	2.5 (2.3 to 2.7)	5.8 (5.4 to 6.2)	6.7 (6.2 to 7.4)
Crohn’s disease	10.4 (10.2 to 10.7)	20.0 (19.6 to 20.4)	27.1 (26.6 to 27.6)
1987–1994	16.3 (15.5 to 17.1)	30.1 (29.9 to 31.8)	40.3 (39.3 to 41.3)
1995–2002	11.9 (11.3 to 12.6)	21.2 (20.4 to 22.0)	27.5 (26.7 to 28.4)
2003–2010	8.5 (7.6 to 8.5)	15.3 (14.7 to 16.0)	20.8 (20.0 to 21.5)
2011–2017	6.7 (6.3 to 7.3)	13.9 (13.0 to 14.7)	16.6 (15.1 to 18.2)
Unclassified IBD	6.3 (5.9 to 6.8)	10.7 (10.2 to 11.3)	14.4 (13.7 to 15.2)
1987–1994	9.0 (7.7 to 10.5)	18.2 (16.4 to 20.1)	25.7 (23.7 to 27.9)
1995–2002	6.7 (5.8 to 7.8)	12.3 (11.1 to 13.7)	16.5 (15.1 to 18.1)
2003–2010	4.8 (4.1 to 5.6)	9.3 (8.4 to 10.3)	13.2 (12.1 to 14.5)
2011–2017	3.7 (3.2 to 4.3)	8.3 (7.3 to 9.4)	9.7 (8.5 to 11.2)

IBD, inflammatory bowel disease.

### Multivariable analyses

Finally, we fitted multivariable Cox proportional hazards models to eliminate mutual confounding between variables that affected the probability of major surgery (table 3). Because findings from univariate models were similar to results from multivariable-adjusted models, we only discuss the multivariable-adjusted results. In patients with UC, the probability of surgical treatment declined by 61%; the HR decreased monotonically and was 0.39 (95% CI 0.36 to 0.42) in patients diagnosed in 2011–2017 compared with those diagnosed in 1987–1994 (p for trend <0.001). The probability of major surgery was slightly but significantly lower in patients diagnosed at ages 20–60 years (table 3). The need for major surgery was more than two times higher in patients with extensive disease, as compared with those with only left-sided colitis (table 3).

**Table 3:** Univariate and multivariate HRs and 95% CIs for major surgery in ulcerative colitis and Crohn’s disease, by time of diagnosis

	Univariate analysis		Multivariate analysis*	
	HR	95% CI	HR	95% CI
Ulcerative colitis				
1987–1994	1.0		1.0	
1995–2002	0.56	0.54 to 0.59	0.59	0.56 to 0.62
2003–2010	0.42	0.40 to 0.44	0.45	0.42 to 0.47
2011–2017	0.33	0.31 to 0.36	0.39	0.36 to 0.42
	p<0.001		p<0.001	
Age at diagnosis				
<20	1.0		1.0	
20–40	0.81	0.76 to 0.86	0.90	0.84 to 0.96
40–60	0.74	0.69 to 0.79	0.85	0.79 to 0.90
>60	0.82	0.77 to 0.89	1.01	0.94 to 1.09
	p<0.001		p<0.001	
Sex				
Female	1.0		1.0	
Male	0.97	0.95 to 1.0	0.95	0.91 to 0.99
Nationality				
Sweden	1.0		1.0	
Norway	0.95	0.92 to 0.99	0.90	0.85 to 0.95
Extent of disease				
Proctitis	1.0		1.0	
Left-sided colitis	0.46	0.42 to 0.51	0.50	0.45 to 0.56
Extensive	2.36	2.21 to 2.51	2.33	2.18 to 2.48
Not defined	1.73	1.61 to 1.87	1.53	1.42 to 1.66
Crohn’s disease				
1987–1994	1.0		1.0	
1995–2002	0.63	0.61 to 0.66	0.81	0.77 to 0.85
2003–2010	0.46	0.43 to 0.48	0.69	0.65 to 0.72
2011–2017	0.39	0.37 to 0.42	0.69	0.65 to 0.75
	p<0.001		p<0.001	
Age at diagnosis				
<20	1.0		1.0	
20–40	1.06	1.01 to 1.12	1.00	0.94 to 1.06
40–60	0.95	0.90 to 1.01	0.92	0.86 to 0.97
>60	0.82	0.76 to 0.88	0.95	0.89 to 1.02
	p<0.001		p<0.001	
Sex				
Female	1.0		1.0	
Male	0.99	0.96 to 1.02	0.96	0.93 to 1.00
Nationality				
Sweden	1.0		1.0	
Norway	0.97	0.94 to 1.01	0.91	0.86 to 0.96
Location of disease				
Terminal ileum	1.0		1.0	
Colon	0.69	0.64 to 0.73	0.74	0.69 to 0.79
Ileocolonic	1.63	1.55 to 1.71	1.29	1.23 to 1.36
Not defined	0.38	0.34 to 0.42	0.43	0.39 to 0.48
Behaviour of disease				
Non-stricturing, non-penetrating	1.0		1.0	
Stricturing	4.19	4.02 to 4.38	3.43	3.28 to 3.59
Penetrating	3.39	3.19 to 3.61	2.81	2.62 to 3.01
Both stricturing and penetrating	6.50	6.09 to 6.94	4.70	4.34 to 5.03
Perianal disease				
No	1.0		1.0	
Yes	1.92	1.84 to 2.00	0.99	0.94 to 1.04

HRs for major surgery during follow-up in a cohort of 128 834 patients with ulcerative colitis and Crohn’s disease.

* Adjusted for year of diagnosis (groups 1987–1994, 1995–2002, 2003–2010 and 2011–2017), age, sex, nationality, extent of disease (UC), behaviour of disease (CD) and perianal disease (CD).

CD, Crohn’s disease; UC, ulcerative colitis.

In patients with CD, the reduction in major surgery over time was lower than for UC, with a 31% decline (HR 0.69, 95% CI 0.65 to 0.75) in patients diagnosed in 2003–2017 compared with those diagnosed in 1987–1994 (p for trend <0.001) (table 3). In contrast to patients with UC, the decline in trends for surgery stopped in 2010 (HR 0.69 for both groups). Age at diagnosis and sex affected probability of surgery only marginally. Patients with ileocolonic disease had significantly 29% higher probability of major surgery than patients with disease affecting only terminal ileum; the probability of surgery was about three to five times higher among patients with stricturing and/or penetrating disease compared with those without (table 3).

## DISCUSSION

In this study of 141 109 patients with IBD followed for 1 395 459 person-years, our most salient findings, supporting our à priori hypothesis, were the substantial, temporal decline in surgical treatment of patients with UC. We also observed a much higher need for surgical treatment in patients with CD with a more modest decline, apparently halting at the beginning of this millennium. Our study further provides information on surgical interventions and diagnoses associated with these procedures. The findings in relation to sex, age at diagnosis and extent of disease are also novel. Important findings from the multivariate analysis are the increased probability of surgery for patients with extensive disease in UC and for patients with stricturing or both stricturing and penetrating diseases in CD. The largely similar results in Norway and Sweden with no statistically significant difference in temporal trends of surgical treatment support the robustness and generalisability of our findings.

The published literature offers only limited data about population-based long-term trends in the need for surgical treatment separately for UC and CD. Our study extends, however, the evidence in the most informative prior study with a similar design undertaken in Denmark, covering the period 1979–2011.^20^ Although the Danish study focused on trends in medical treatment, it also documented a more modest decline in surgical treatment of UC and generally higher rates of surgery for CD than we found. This difference may arise because follow-up ended in 2011, before the positive impact of advanced therapies became evident. Advantages of our study include its much larger size, analysis of 6 more years of follow-up and the comparison between two countries. We further required two IBD diagnoses for inclusion, to increase the positive predictive value while the former Danish study only required one diagnosis of IBD for inclusion.^20^ A French study of therapeutic management, including surgery, from 2009 to 2014, showed numbers of cumulative probability of major surgery that were similar to ours for the corresponding, more recent, time period.^21^ The study was large, 210 001 patients, but had a shorter follow-up time (median 4.9 years). The majority (if not all) of other studies on the topic did not censor patients at date of CRC diagnosis, which we think is essential to isolate surgery due to IBD and not CRC in these patients.^15^,^1820^

At least two factors could explain the more modest decline in surgical treatment among patients with CD compared with UC. First, the use of effective biologic treatments might have penetrated management of CD less effectively than UC, although previous studies show similar usage of biologic treatment in CD and UC.^21 28^ Further studies might test this hypothesis by individual linkage to the prescription registers now available in both Norway and Sweden. Second, novel drugs may be less effective in reducing the need for surgical treatment in CD than in UC. In the era of biologic treatment, up to 30% of CD patients never achieve good disease control,^11^ and CD patients with inflammation located to the ileum are thought not to respond as well to TNF-alpha inhibitors compared with CD located elsewhere.^41^,^43^ This reflects the larger need for surgical treatment in CD patients with ileal or ileocolonic disease compared with those with colonic involvement that we saw in our study. It has also been proposed that in CD, TNF-alpha inhibitors have anti-inflammatory effects but do not stop the fibrosis initiated by inflammation, leading to excessive repair processes, with fibrosis and strictures.^44^

Our study shows that total colectomy is by far the most common procedure in UC, potentially a curative treatment for these patients. In the situation of emergency surgery of acute severe UC, subtotal colectomy with reconstruction in a two-step or three-step approach is advised, while in refractory UC, there is currently less consensus regarding the surgical approach.^14^ In immunosuppressed patients, there is an increased risk for anastomotic failure, which the surgeons need to consider when choosing the surgical method.^45 46^ In CD, ileocaecal resection is the most common procedure, and studies have shown that this therapy is a good alternative to biologic agents for patients with ileocaecal involvement.^47 48^

Strengths of our study include its large size, population-based design and virtually complete follow-up in nationwide high-quality registers covering the entire period when new biologic drugs were introduced. The public healthcare systems in Norway and Sweden guarantee all residents equal access to competent inpatient care. We also aimed to overcome or reduce some of the fundamental methodological challenges in studies of IBD.^49^ In particular, we tried to define the date of disease onset as precisely as possible and to distinguish incident from prevalent cases of IBD. Finally, we required two similar diagnoses to optimise the predictive value when patients were classified as having UC or CD.

**Figure 2:**
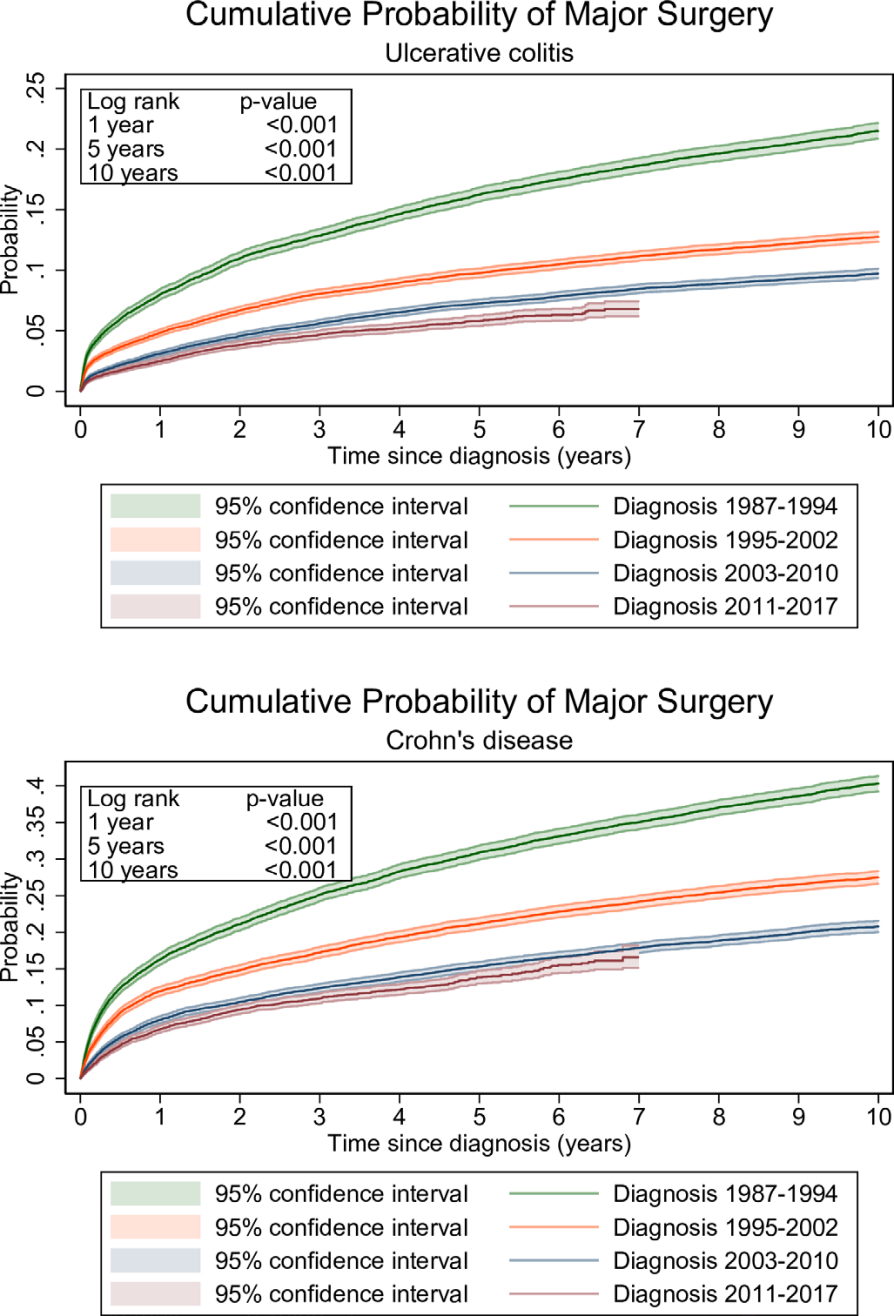
Cumulative probability of first major surgery in patients with ulcerative colitis and Crohn’s disease. Blue line diagnosed 1987–1994, red line 1995–2002, green line 2003–2010, yellow line 2011–2017. P-value for homogeneity performed by log-rank test, p-value compares rates within the first year after diagnosis, from 1 to 5 years and 6–10 years after diagnosis. For IBD-U, see online supplemental figure 7. IBD-U, unclassified inflammatory bowel disease.

Our study also has limitations. First, left censoring in enrolment to our analytic cohort likely entails that some patients identified early had prevalent rather than incident disease. Because the need for surgical treatment is highest in the first years following diagnosis (figure 2, IBD-U see online supplemental figure 7), this might have biased quantification of surgical treatment downward particularly during the first period of enrolment (1987–1994). Therefore, temporal trends might indeed have been slightly underestimated. Second, from 2001, we identified patients in Sweden also from the outpatient register who had not required inpatient care. These patients were likely to have less severe disease with a lower need for surgical treatment, thereby potentially augmenting the downward temporal trend for surgical treatment. The similar results in Norway and Sweden indicate, however, that such a bias, if any, is small. Finally, we were unable to analyse the most recent time period because we did not have any data after 2015 in Norway and 2017 in Sweden. Hence, future research is needed to clarify if the temporal trends, particularly in UC, have continued beyond 2017.

In conclusion, the management of IBD has been dramatically transformed during the last decades, particularly in patients with UC, although surgery still plays an important role both in elective care and acute treatment. The decreasing trend for mutilating surgical treatment—probably predominantly attributable to the advent of improved pharmacological therapy—is indeed a success story not only for the individual patients with IBD but also for the less burdened healthcare system. Future research should investigate if optimised treatment with biologic drugs can further reduce the need for surgical intervention in patients with IBD.

## Supplementary material

10.1136/bmjgast-2025-001828 online supplemental file 1

10.1136/bmjgast-2025-001828 online supplemental file 2

## Data Availability

Data are available upon reasonable request.
