# Supplementary material for: Temporal trends in surgical treatment of inflammatory bowel disease following introduction of biological drugs in Norway and Sweden
Source: BMJ Open Gastroenterol. 2025 Jun 19;12(1):e001828. doi: 10.1136/bmjgast-2025-001828 (PMC12182004; doi:10.1136/bmjgast-2025-001828)
Supplement: online supplemental file 1 [file bmjgast-12-1-s001.pdf]

Supplementary

Table 1- ICD Codes for IBD diagnoses 2
Table 2- Codes for Surgical Procedures Sweden 3
Table 3- Codes for Surgical Procedures Norway 4
Table 4- Codes for Associated Diagnoses 5
Table 5- Classification of Extent of Ulcerative Colitis and Location and Behavior of Crohn's Disease 6
Table 6. Incidence Rates of Surgery per 1000-person years in UC patients at 0-1 years, 1-5 years, 5-10 years and >10 years. 7
Table 7. Incidence Rates of Surgery per 1000 person-years in CD patients at 0-1 years, 1-5 years, 5-10 years and >10 years. 7
Table 8- Distribution of Numbers and Cases- IBD-U 8
Table 9. Surgical Procedures per 1000 Patients with Prevalent Ulcerative Colitis and Crohn's Disease, by type of Procedure. 9
Table 10- Age at Major Surgery 10
Figure 1: Flowchart of the Swedish IBD Cohort Included in Main Analysis 11
Figure 2: Flowchart of the Norwegian IBD Cohort Included in Main Analysis 12
Figure 3. Distribution of Time Between First and Second Registered IBD Diagnosis. 13
Figure 4. Indication for Major Surgery in Patients with Unclassified IBD. 14
Figure 5. Surgical Procedures per 1000 Patients with Prevalent Ulcerative Colitis, Divided by type of Procedure 15
Figure 6. Surgical Procedures per 1000 Patients with Prevalent Crohn's Disease Divided by type of Procedure. 16
Figure 7. Cumulative Probability of First Major Surgery in Patients with Unclassified IBD. 17

Table 1- ICD Codes for IBD diagnoses		
	ICD 9	ICD 10
UC	556, 556X	K51, K510, K512, K513, K514, K515, K518, K519
CD	555, 555,0, 555A, 555B, 555C, 555X	K50, K500, K501, K508, K509
IBD-U	558	K523

Table 2- Codes for Surgical Procedures Sweden		
Surgical procedures	Classification of surgical procedures 6th edition 1963-1996	Swedish version of NOMESCO Classification of surgical procedures version 1.9
Local procedure	4600, 4601, 4740, 4610, 4611, 4741, 4042, 4043, 4048	JFA00, JFA01, JFA17, JFA60, JFA63, JFA65, JFA70, JFA71, JFA73, JFA74, JFA76, JFA80, JFA81, JFA86, JFA96, JFA97, JFA98
Small bowel resection	4630, 4631	JFB00, JFB01, JFB10, JFB13
Anastomosis	4730, 4731, 4734, 4732, 4735, 4739	JFC00, JFC01, JFC10, JFC11, JFC20, JFC21, JFC30, JFC40, JFC41, JFC50, JFC51
Stoma	4600, 4661, 4662, 4663, 4700, 4671, 4668, 4792	JFF00, JFF01, JFF10, JFF11, JFF13, JFF20, JFF21, JFF23, JFF24, JFF26, JFF27, JFF30, JFF31, JFF40, JFF41, JFF50, JFF51, JFF60, JFF96, JFF97
Distal colectomy	4644, 4640	JFB46, JFB47, JFB60, JFB61, JFB53, JFB54, JFB43, JFB44
Proximal colectomy	4641, 4643	JFB30, JFB31, JFB40, JFB41, JFB33, JFB34,
Total colectomy	4650, 4651	JFH00, JFH01, JFH10, JFH11, JFH96
Proctocolectomy	4652, 4653, 4654	JFH20, JFH21, JFH30, JFH31, JFH33, JFH40
Proctectomy	4820, 4821, 4822, 4828, 4823, 4829	JGA76, JGB00, JGB01, JGB10, JGB11, JGB20, JGB30, JGB31, JGB40, JGB50, JGB60, JGB96, JGB97, JGB03, JGB04, JGB33, JGB34, JGB36, JGB61
Ileocecal resection	4642, 4648	JFB20, JFB21
Other	4649	JFB50, JFB51, JFB63, JFB64, JFB96, JFB97, JFW96, JFW97, JFW98, JGW96, JGW97

Table 3- Codes for Surgical Procedures Norway

Surgical procedures	SIFF code (Statens helsetilsyn og Folkehelsa) 2nd edition 1992, 3rd edition 1995	Norwegian version of NOMESCO Classification of surgical procedures NKPK, NCSP; a common Nordic classification system of surgical procedures and interventions
Local procedure	4600, 4601, 4602, 4720, 4630, 4740, 4610, 4604, 4611, 4603, 4741, 4042, 4043, 4048	JFA00, JFA01, JFA17, JFA60, JFA63, JFA65, JFA70, JFA71, JFA73, JFA74, JFA76, JFA80, JFA81, JFA86, JFA96, JFA97, JFA98
Small bowel resection	4630, 4631, 3724, 4722, 4620, 4621, 4622	JFB00, JFB01, JFB10, JFB13
Anastomosis	4730, 4731, 4725, 4726, 4734, 4732, 4735, 4733, 4727, 4739	JFC00, JFC01, JFC10, JFC11, JFC20, JFC21, JFC30, JFC40, JFC41, JFC50, JFC51
Stoma	4600, 4661, 4662, 4663, 4632, 4700, 4630, 4631, 4671, 4668, 4660, 4792	JFF00, JFF01, JFF10, JFF11, JFF13, JFF20, JFF21, JFF23, JFF24, JFF26, JFF27, JFF30, JFF31, JFF40, JFF41, JFF50, JFF51, JFF60, JFF96, JFF97
Distal colectomy	4644, 4640, 4645	JFB46, JFB47, JFB60, JFB61, JFB53, JFB54, JFB43, JFB44
Proximal colectomy	4641, 4643, 4646	JFB30, JFB31, JFB 40, JFB 41, JFB33, JFB34
Total colectomy	4650, 4651, 4659	JFH00, JFH01, JFH10, JFH11, JFH96
Proctocolectomy	4652, 4653, 4654. 4655	JFH20, JFH21, JFH30, JFH31, JFH33, JFH40
Proctectomy	4820, 4821, 4822, 4823, 4824, 4829	JGA76, JGB00, JGB01, JGB03, JGB04, JGB06, JGB07, JGB10, JGB11, JGB20, JGB21, JGB30, JGB31, JGB40, JGB50, JGB60, JGB96, JGB97, JGB33, JGB34, JGB36, JGB61
Ileocecal resection	4642	JFB20, JFB21
Other	4649, 4673, 4723, 4724, 4049, 4721, 4629	JFB50, JFB51, JFB63, JFB64, JFB96, JFB97

Table 4- Codes for Associated Diagnoses		
Diagnosis code	ICD 9	ICD 10
Benign tumor	211D, 211E, 211X	D12, D120, D122, D123, D124, D125, D126, D127, D128
Cancer in situ (CIS)	230D, 230E, 230H	D01, D010, D011, D012, D014
Colorectal cancer (CRC)*	153, 153A, 153B, 153C, 153D, 153E, 153G, 153H, 153W, 153X, 154, 154A, 154B	C18, C180, C182, C183, C184, C185, C186, C187, C188, C189, C19, C20
Obstruction- incl ileus	560, 560A, 560B, 560C, 560W, 560X	K56, K561, K560, K562, K565, K567
Mesenteric thromboembolism	557, 557A, 557B, 557X	K550
Inflammatory bowel disease (IBD)	555, 555A, 555B, 555C, 555X, 556, 556X, 558	K50, K500, K501, K508, K509, K51, K510, K512, K513, K514, K515, K518, K519, K523
Functional disorders	564, 564B, 564X	K52, K58, K59, K599
Fissures/fistulas	565A, 565B	K60, K600, K601, K602, K603, K604, K605
Peritonitis/ acute inflammation	567, 567C, 567W, 567X, 568, 569W	K65, K650, K658, K659, K630, K632
Stomal dysfunction	569G	K914
* CRC diagnosis used for censoring patients in survival analysis.		

Table 5- Classification of Extent of Ulcerative Colitis and Location and Behavior of Crohn's Disease

		Diagnostic codes ICD 10, ICD 9	Procedure code
**Ulcerative colitis**	**Extent**		
E1	Ulcerative proctitis	K51.2,	
E2	Left-sided UC	K51.3, K51.5	
E3	Extensive UC	K510, K511	
E0	Extent not defined	K51.4, K51.9, K51.8	
**Crohn's Disease**	**Location of disease**		
L1	Terminal ileum	K50.0, 555.0/555A	
L2	Colon	K50.1, 555.1/555B	
L3	Ileocolonic	K50.8, 555.2/555C/555AB	
L0	Not defined	K50.9, 555.9/555X	
	**Behavior of disease**	Code of CD and any of the following codes:	*Code of CD and any of the following procedure codes*
B1	Non stricturing, non penetrating	None of ICD codes for B2 or B3	
B2	Stricturing	K56.5, K56.6, K56.7, K62.4, 560.8/560W, 560.9/560X	
B3	Penetrating	K63.0, K63.2, K31.6, N82.3, N82.4, 537.4/537E, 569.6/569F, 596.1/596B, 619.1/619B, 629.8	JHD20, JHD30, JHD33, JHD50, JHD60, JHD63, JFA76, JFA86, 4603/4611, 4962/4922
B4	Both stricturing and penetrating	ICD codes for both B2 and B3	
	**Perianal disease**		
	Yes	K60.3, K60.4, K60.5, K61.0, K61.1, K61.2, K61.3, K61.4, K62.4, 565, 565.0, 565.1,	JHD20, JHD30, JHD33, JHD50, JHD60, JHD63, JHA00, JHA20, JHW96, 4962

E= extent, UC= Ulcerative colitis, L=location, B=behavior, CD= Crohn's disease

Table 6. Incidence Rates of Surgery per 1000-person years in UC patients at 0-1 years, 1-5 years, 5-10 years and >10 years.

Estimated person-time and incidence rates

Cohort	person-years	Failures	Rate	[95% conf.	interval]
(0 - 1]	81210.43	3706	45.634533	44.1887	47.12767
(1 - 5]	274145.15	3868	14.109314	13.67161	14.56104
(5 - 10]	252372.01	1961	7.7702753	7.433864	8.12191
> 10	305238.46	1652	5.412162	5.157371	5.679541
Total	912966.05	11187	12.253468	12.02849	12.48265

Table 7. Incidence Rates of Surgery per 1000 person-years in CD patients at 0-1 years, 1-5 years, 5-10 years and >10 years.

Estimated person-time and incidence rates

Cohort	person-years	Failures	Rate	[95% conf.	interval]
(0 - 1]	38615.688	4371	113.19234	109.886	116.5982
(1 - 5]	121173.21	3481	28.727472	27.78883	29.69782
(5 - 10]	102501.93	1931	18.83867	18.01689	19.69794
> 10	115018.01	1524	13.250099	12.60129	13.93232
Total	377308.83	11307	29.967494	29.42019	30.52498

Table 8- Distribution of Numbers and Cases- IBD-U	**Number (colectomies)**	**Number (colectomies)**
**Year of diagnosis**	**Sweden**	**Norway**
**1987-1994**	**1,051 (374)**	**666 (191)**
**1995-2002**	**1,417 (303)**	**973 (160)**
**2003-2010**	**2,453 (287)**	**1,133 (152)**
**2011-2015**	**3,257 (209)**	**1,425 (85)**
**Full cohort**	**8,178 (1,173)**	**4,197 (588)**

Table 11- Distribution of numbers and cases- IBDU

IBD-U, unclassified Inflammatory Bowel Disease

Table 9. Surgical Procedures per 1000 Patients with Prevalent Ulcerative Colitis and Crohn's Disease, by type of Procedure.

	Local		Small bowel resection		Anastomosis		Proximal colectomy		Distal colectomy		Total colectomy		Proctocolectomy		Proctectomy		Ileocecal resection		Other	
	**UC**	**CD**	**UC**	**CD**	**UC**	**CD**	**UC**	**CD**	**UC**	**CD**	**UC**	**CD**	**UC**	**CD**	**UC**	**CD**	**UC**	**CD**	**UC**	**CD**
1987	4.03	16.25	4.03	84.52	4.71	10.56	2.02	21.94	2.02	10.16	42.69	19.91	27.90	4.47	14.45	11.38	0.34	79.24	2.02	19.91
1988	1.90	7.03	1.15	39.67	0.57	3.40	0.76	11.56	0.57	6.35	31.32	12.47	14.71	4.76	5.54	7.03	0.38	48.06	0.57	10.43
1989	6.12	11.08	1.39	26.40	0.56	2.77	0.70	7.17	1.11	5.38	23.51	9.94	10.15	3.75	4.03	4.07	0.14	38.79	0.28	4.73
1990	5.63	8.58	1.13	15.31	0.11	3.56	0.34	3.69	1.01	3.30	18.25	9.37	7.32	4.35	3.04	3.03	0.23	26.25	0.34	2.24
1991	4.73	6.57	0.47	11.13	0.19	3.23	0.28	3.56	0.85	3.00	18.63	8.57	7.75	2.56	2.46	1.89	0.47	23.92	0.28	2.11
1992	4.63	7.99	0.65	9.98	0.24	0.95	0.73	2.85	1.22	2.76	15.04	6.65	6.34	2.85	1.38	1.24	0.49	21.87	0.57	2.00
1993	3.58	5.52	0.49	7.91	0.21	1.32	0.49	2.97	0.91	1.98	15.71	5.85	6.24	1.73	1.54	1.98	0.21	18.95	0.63	1.32
1994	2.72	5.10	0.31	6.27	0.06	0.95	0.19	2.41	1.05	1.97	15.11	5.25	5.02	2.26	1.48	1.39	0.37	14.29	0.81	0.95
1995	3.05	4.56	0.17	4.75	0.11	0.72	0.39	1.69	0.72	1.82	14.47	5.14	5.77	2.80	1.33	1.50	0.33	16.15	0.17	0.65
1996	2.60	4.88	0.25	4.47	0.25	1.00	0.45	1.53	0.85	1.53	13.75	5.88	4.60	2.53	1.15	1.06	0.20	12.05	0.40	1.00
1997	1.59	2.62	0.22	3.21	0.13	0.32	0.53	3.75	0.88	2.09	11.71	5.03	3.54	1.93	1.06	1.34	0	8.89	0.53	1.02
1998	1.37	3.16	0.55	3.70	0.12	0.15	0.47	4.09	0.82	1.75	12.00	3.99	3.18	1.85	1.25	0.73	0.27	7.88	0.43	1.12
1999	0.68	2.02	0.50	2.47	0.21	0.45	0.71	3.05	0.79	1.75	11.30	4.17	2.57	1.93	1.00	1.21	0.11	7.71	0.43	0.49
2000	0.49	1.63	0.36	1.79	0.03	0.04	0.59	2.50	0.79	0.71	8.76	4.13	2.49	1.71	0.98	0.50	0.16	6.68	0.52	0.67
2001	0.42	1.26	0.17	1.93	0.17	0.26	0.47	2.64	0.47	0.37	7.83	3.57	2.44	1.71	0.61	0.59	0.17	6.73	0.33	0.74
2002	0.45	1.07	0.30	2.28	0.10	0.07	0.53	2.41	0.30	0.90	6.72	3.31	2.26	1.72	0.48	0.69	0.18	5.59	0.35	0.55
2003	0.28	1.04	0.12	1.88	0.16	0.16	0.42	1.78	0.56	1.10	6.40	3.01	2.09	1.49	0.59	0.52	0.05	5.02	0.40	0.45
2004	0.26	1.19	0.15	1.65	0.15	0.15	0.53	2.02	0.48	0.95	5.48	3.06	1.74	1.28	048	0.61	0.07	4.31	0.48	0.70
2005	0.50	0.96	0.33	1.77	0.19	0.26	0.58	1.86	0.46	0.99	5.01	2.70	1.37	0.99	0.56	0.64	0.15	4.62	0.50	0.49
2006	0.31	0.86	0.30	1.72	0.12	0.19	0.37	1.36	0.51	0.83	5.10	2.38	1.28	0.89	0.63	0.58	0.14	3.18	0.37	0.36
2007	0.47	1.16	0.26	1.40	0.11	0.29	0.36	1.16	0.45	0.77	4.27	2.25	1.07	0.71	0.51	0.53	0.19	3.94	0.32	0.45
2008	0.52	0.88	0.21	1.41	0.12	0.23	0.50	1.34	0.53	0.88	4.43	2.12	0.92	0.66	0.60	0.28	0.12	3.23	0.32	0.35
2009	0.32	1.07	0.32	1.24	0.09	0.17	0.55	1.24	0.68	0.97	4.41	2.57	0.85	0.36	0.49	0.44	0.17	3.35	0.32	0.65
2010	0.31	0.93	0.25	1.54	0.11	0.28	0.38	1.19	0.37	0.70	4.27	2.38	0.77	0.61	0.30	0.26	0.08	3.43	0.38	0.37
2011	0.27	0.61	0.22	0.99	0.14	0.27	0.33	1.10	0.52	0.90	4.19	2.49	0.80	0.56	0.36	0.25	0.16	3.77	0.39	0.79
2012	0.29	0.74	0.27	0.85	0.11	0.17	0.47	1.37	0.33	0.61	4.00	2.19	0.62	0.46	0.24	0.24	0.11	3.19	0.20	0.41
2013	0.26	0.48	0.34	1.18	0.12	0.23	0.44	0.84	0.47	0.76	3.78	2.37	0.78	0.46	0.46	0.29	0.09	3.02	0.24	0.42
2014	0.19	0.57	0.27	0.87	0.06	0.14	0.37	0.95	0.47	0.75	3.51	2.39	0.74	0.45	0.24	0.26	0.14	3.30	0.19	0.32
2015	0.27	0.41	0.27	0.88	0.06	0.18	0.51	0.86	0.55	0.69	3.02	2.12	0.36	0.18	0.46	0.49	0.14	2.77	0.21	0.31
2016	0.21	0.56	0.27	1.73	0.12	0.26	0.41	1.13	0.66	0.71	3.26	1.01	0.31	0.11	0.35	0.26	0.14	5.63	0.14	0.64
2017	0.18	0.84	0.36	2.11	0.16	0.29	0.48	1.46	0.60	0.66	2.78	0.91	0.34	0.11	0.30	0.33	0.14	5.32	0.14	0.51

Table 10- Age at Major Surgery	Mean age (±SD)	
	UC	CD
1987-1994	45.7 (±16.8)	41.8 (±16.0)
1995-2002	48.6 (±18.4)	43.3 (±17.5)
2003-2010	49.1 (±19.5)	43.7 (±19.1)
2011-2017	49.6 (±20.7)	45.1 (±19.9)
Full cohort	47.7 (±18.3)	42.9 (±17.5)

Table 6. Mean age at surgery, subgroups by year of diagnosis. SD, standard deviation.

Figure 1: Flowchart of the Swedish IBD Cohort Included in Main Analysis

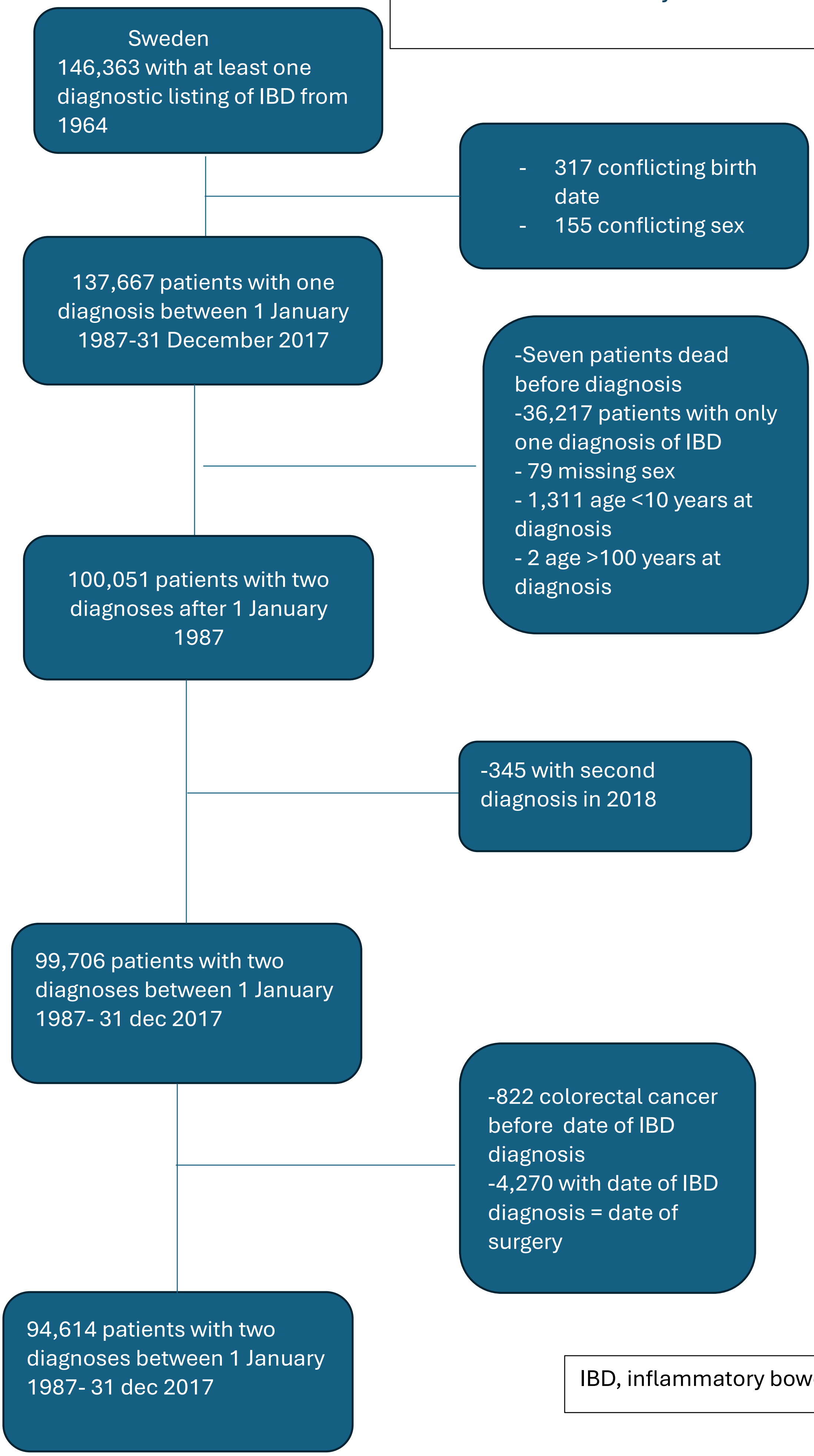

IBD, inflammatory bowel disease.

Figure 2: Flowchart of the Norwegian IBD Cohort Included in Main Analysis

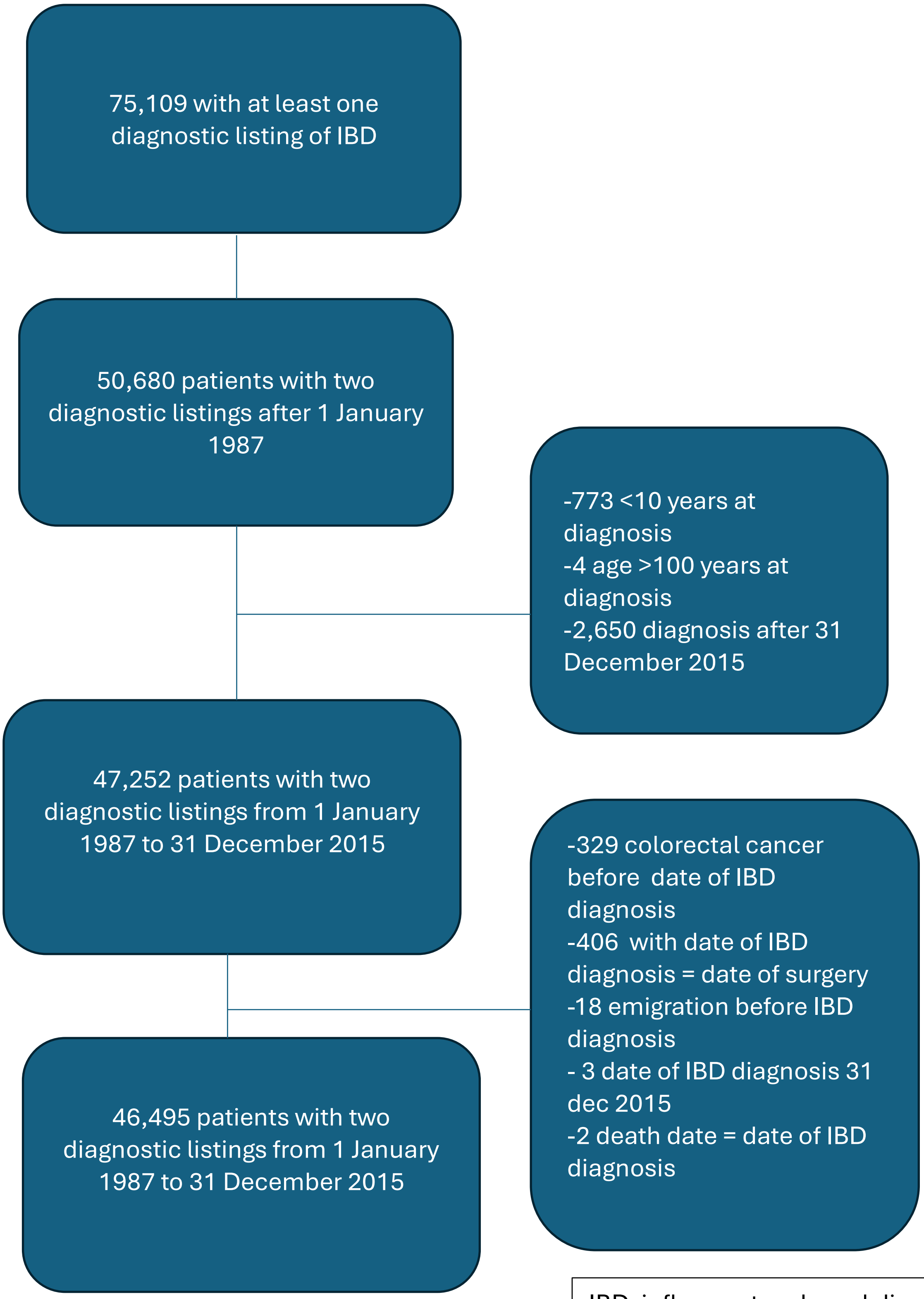

IBD, inflammatory bowel disease.

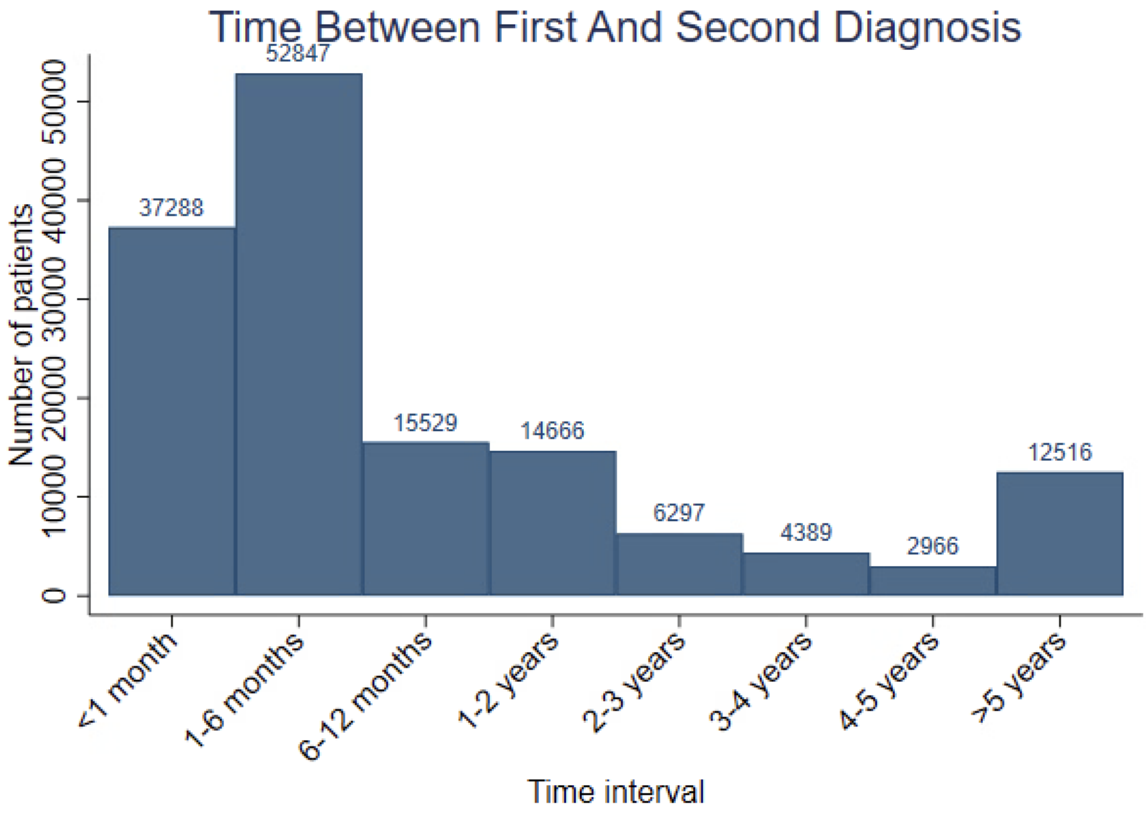

Figure 3. Distribution of Time Between First and Second Registered IBD Diagnosis.

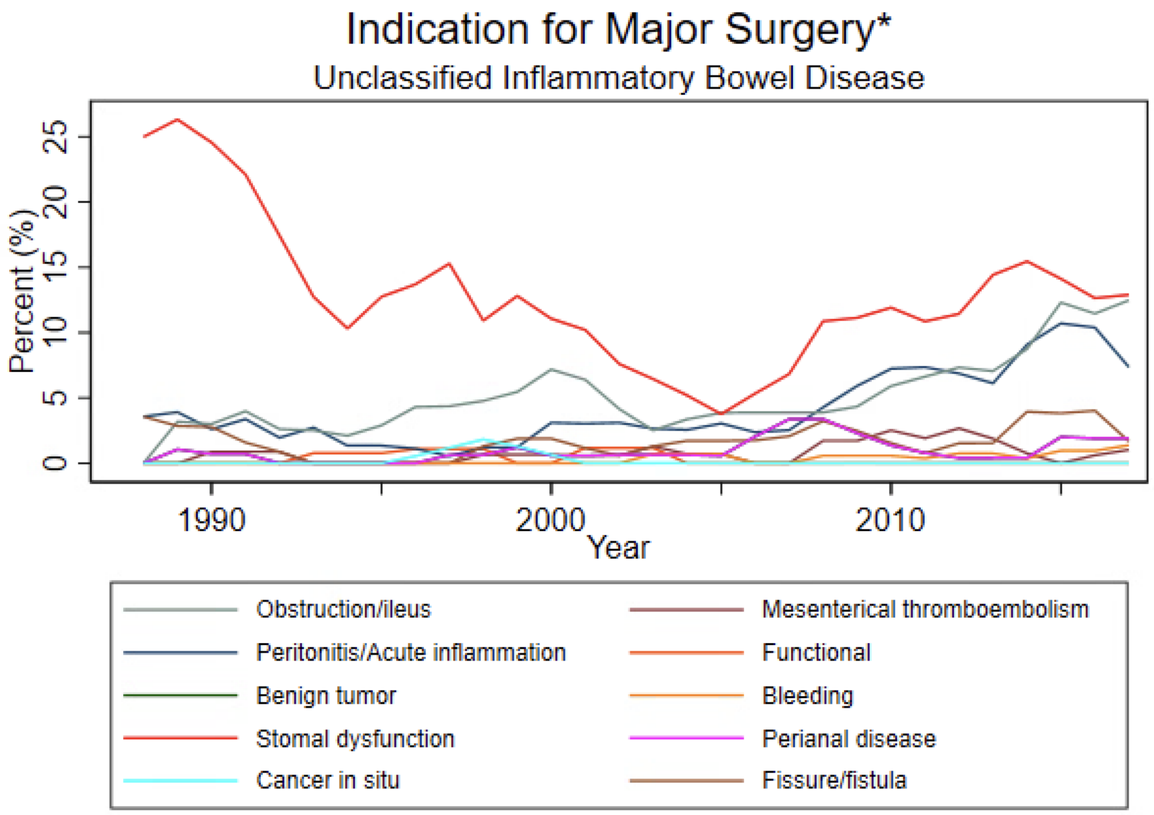

Figure 4. Indication for Major Surgery in Patients with Unclassified IBD.

Percentage of procedures with specific diagnoses, by year. IBD diagnosis excluded. Smoothed curve over average of 3 years.
*ICD codes registered at the date of surgery was used as a proxy for indication.

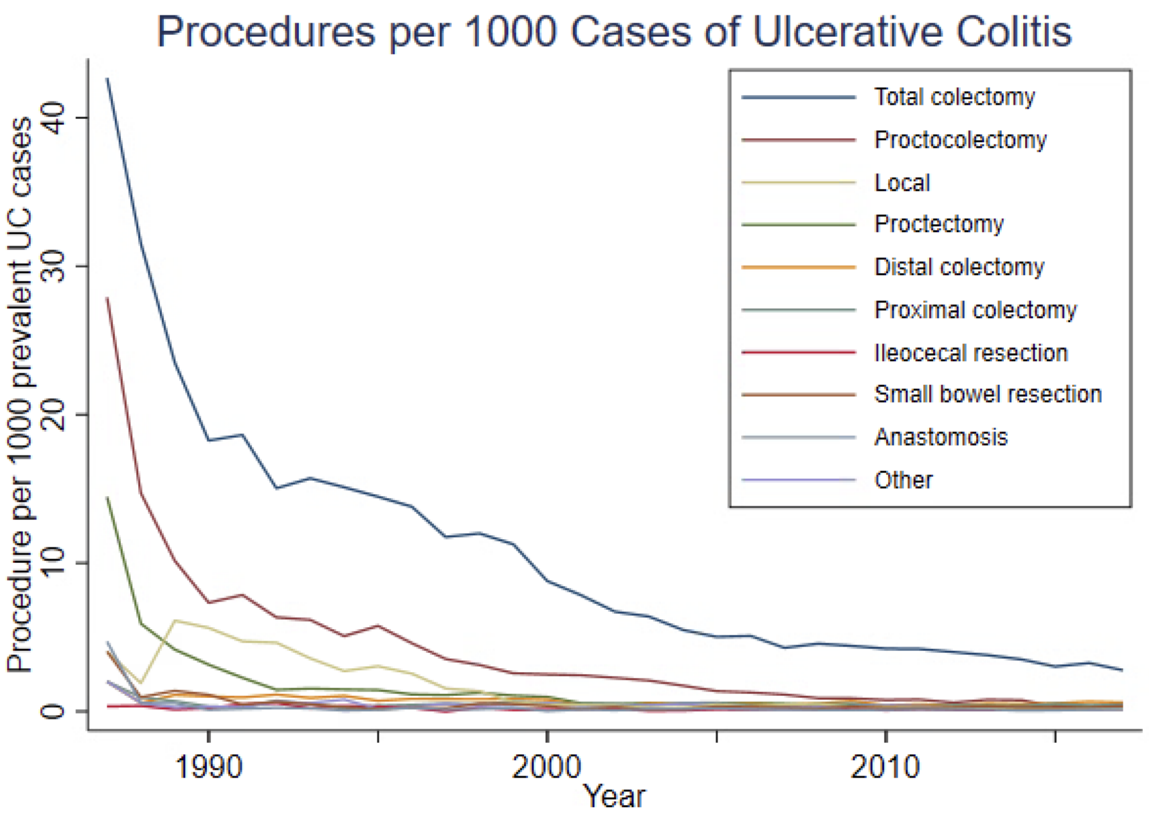

Figure 5. Surgical Procedures per 1000 Patients with Prevalent Ulcerative Colitis, Divided by type of Procedure

UC- Ulcerative colitis

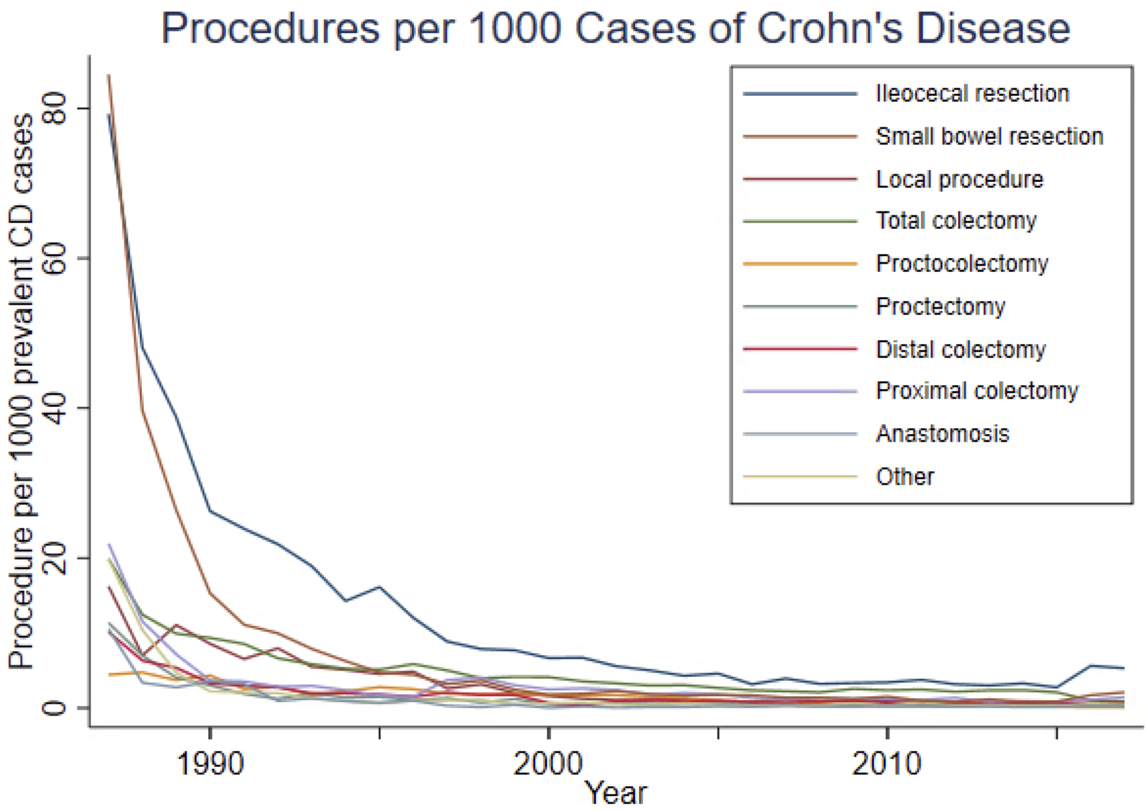

Figure 6. Surgical Procedures per 1000 Patients with Prevalent Crohn's Disease Divided by type of Procedure.

CD- Crohn's disease

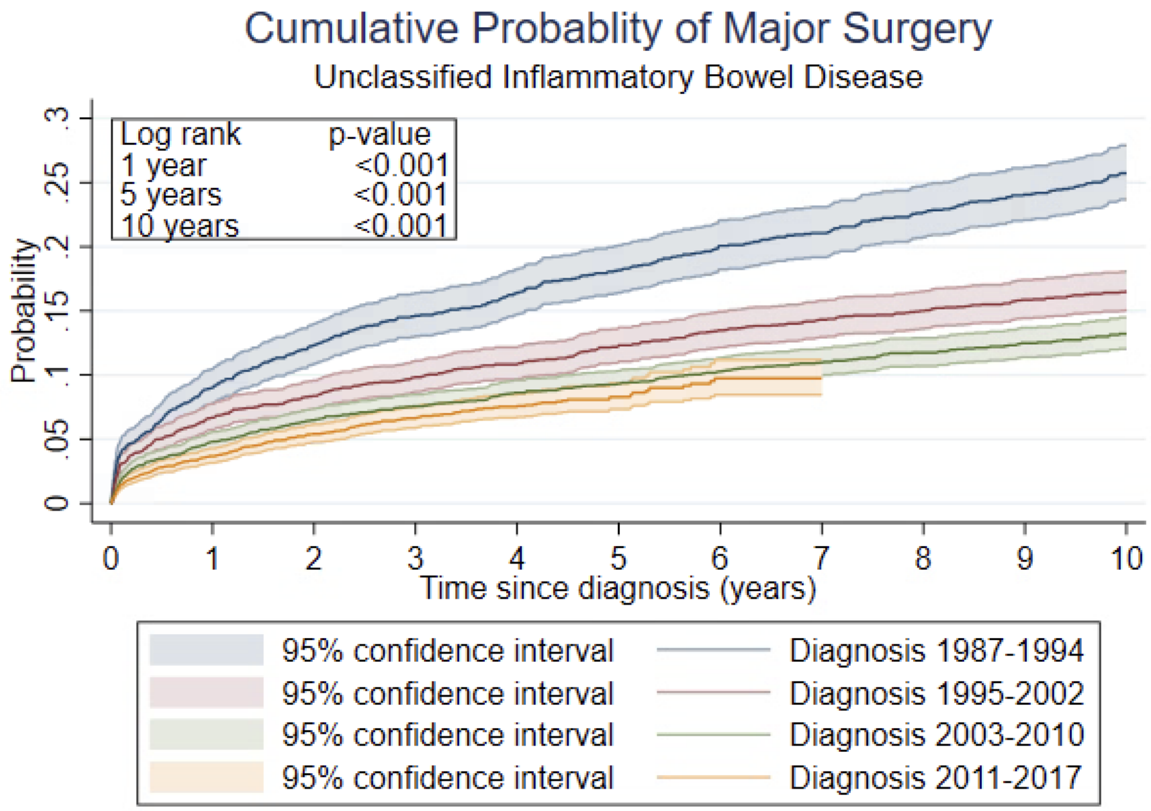

Figure 7. Cumulative Probability of First Major Surgery in Patients with Unclassified IBD. Blue line diagnosed 1987-1994, red line 1995-2002, green line 2003-2010, yellow line 2011-2017. p Value for homogeneity performed by log rank test, p value compares rates within first year after diagnosis, from 1–5 years and 6–10 years after diagnosis.
